# Supplementary material for: Conformational dynamics, RNA binding, and phase separation regulate the multifunctionality of rabies virus P protein
Source: Nat Commun. 2025 Oct 29;16:9491. doi: 10.1038/s41467-025-65223-y (PMC12589655; doi:10.1038/s41467-025-65223-y)
Supplement: Supplementary file 1 — Supplementary Information [file 41467_2025_65223_MOESM1_ESM.pdf]

Supplementary Information for:

Conformational dynamics, RNA binding, and phase separation regulate the multifunctionality of rabies virus P protein

Stephen M. Rawlinson[1,*‡], Shatabdi Chakraborty[2,3*], Ashish Sethi[2,3,4,*], Cassandra T. David[1], Angela R. Harrison[5], Lauren E. Bird[1,6], Ashley M. Rozario[7], Sanjeev Uthishtran[8,9], Katie Ardipradja[10], Tianyue Zhao[1], Sibil Oksayan[5], David A. Jans[5], Ching-Seng Ang[3], Zhi Hui Lu[2,3], Fei Yan[2,3], Nicholas A. Williamson[3], Senthil Arumugam[8,9], Vinod Sundaramoorthy[10,11], Toby D. M. Bell[12], Paul R. Gooley[2,3‡], Gregory W. Moseley[1‡]

1 Department of Microbiology, Biomedicine Discovery Institute, Monash University, Clayton, VIC 3800, Australia

2 Department of Biochemistry and Pharmacology, University of Melbourne, Parkville, VIC 3010, Australia

3 Bio21 Molecular Science and Biotechnology Institute, University of Melbourne, Parkville, VIC 3010, Australia

4 Australian Nuclear Science and Technology Organisation, Australian Synchrotron, 800 Blackburn Rd, Clayton, VIC 3168, Australia

5 Department of Biochemistry and Molecular Biology, Biomedicine Discovery Institute, Monash University, Melbourne, VIC 3800, Australia

6 Department of Microbiology and Immunology at the Peter Doherty Institute for Infection and Immunity, The University of Melbourne, Melbourne, VIC 3000, Australia

7 La Trobe Institute for Molecular Science, La Trobe University, Bendigo, VIC 3552, Australia

8 Department of Anatomy and Developmental Biology, Biomedicine Discovery Institute, Monash University, Melbourne, Victoria, Australia

9 European Molecular Biological Laboratory Australia (EMBL Australia), Monash University, Clayton/Melbourne, Victoria, Australia

10 Commonwealth Scientific and Industrial Research Organisation (CSIRO), Australian Centre for Disease Preparedness (ACDP), East Geelong, Victoria, Australia

11 School of Medicine, Deakin University, Geelong, Victoria, Australia

12 School of Chemistry, Monash University, Clayton, Victoria, 3800, Australia

Correspondence:

‡ Correspondence to: G.W.M. (greg.moseley@monash.edu); P.R.G. (prg@unimelb.edu.au); S.M.R. (stephen.rawlinson@monash.edu)

Equal contribution:

* S.M.R., A.S. and S.C. contributed equally to this work.

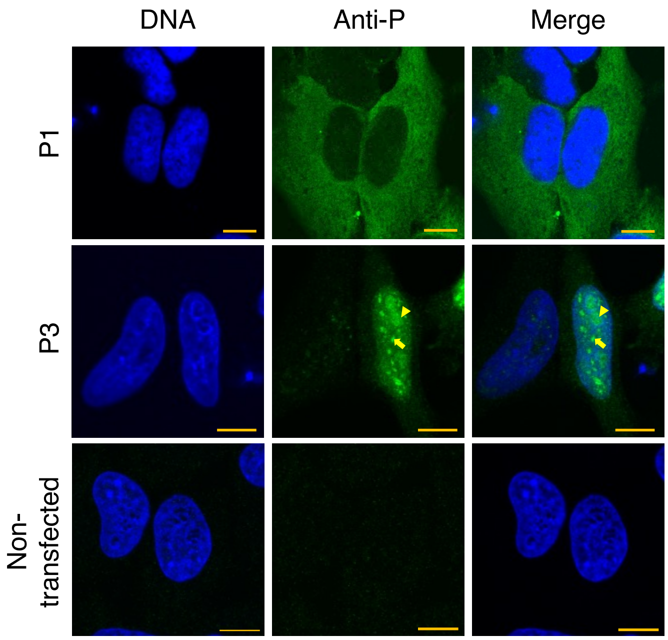

Supplementary Figure 1. Non-fused P3 localizes to nucleoli and NBs in HeLa cells. (A) HeLa cells transfected to express untagged CVS-11 P1 or P3, or non-transfected control. Cells were fixed at 20 h post-transfection (p.t.) and immunostained using anti-P protein antibody. Yellow arrowhead indicates P3 within nucleoli, and yellow arrow indicates P3-associated NBs. Images representative of cells in ≥ 18 fields of view, from two independent assays. Scale bar, 10 μm (orange).

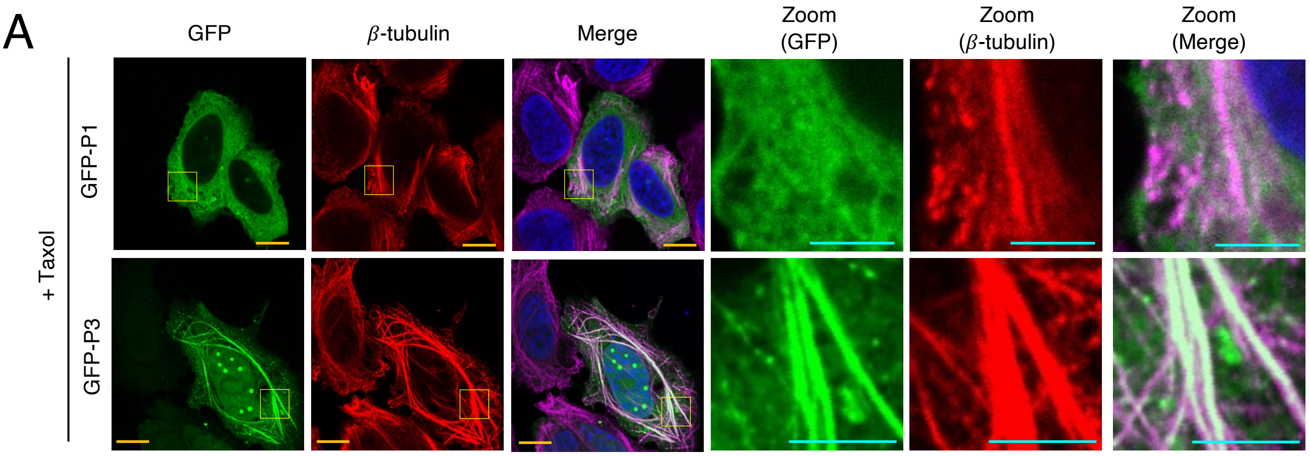

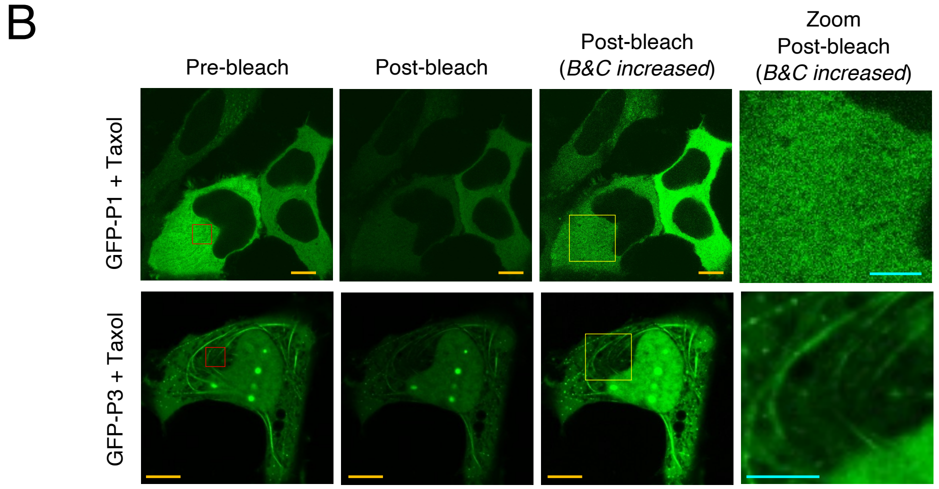

Supplementary Figure 2. GFP-P1 does not associate with microtubules.

(A) HeLa cells expressing GFP-P1 or GFP-P3 were treated with Taxol prior to fixation and immunostaining for β-tubulin. Representative CLSM images are shown. Hoechst (blue) was used to visualize nuclei, and merged images show GFP-P1/P3 (green), DNA (blue) and β-tubulin (magenta). Yellow boxes indicate magnified regions displayed in the right panels (Zoom).

(B) Live-cell CLSM imaging of HeLa cells expressing GFP-P1 or GFP-P3 following Taxol treatment. Cells were imaged before photobleaching (pre-bleach), then repeatedly bleached in a defined cytosolic region (red box) for 5–10 min to reduce cytosolic GFP fluorescence (post-bleach). Pre- and post-bleach images were acquired using identical microscope settings. Brightness and contrast were digitally adjusted to enable visualization of cytosolic fluorescence (B&C increased). Yellow boxes indicate regions shown at higher magnification in the right Zoom panels.

Scale bars: 10 μm (orange) for full-field images; 5 μm (light blue) for zoomed regions (applies to all images in A and B).

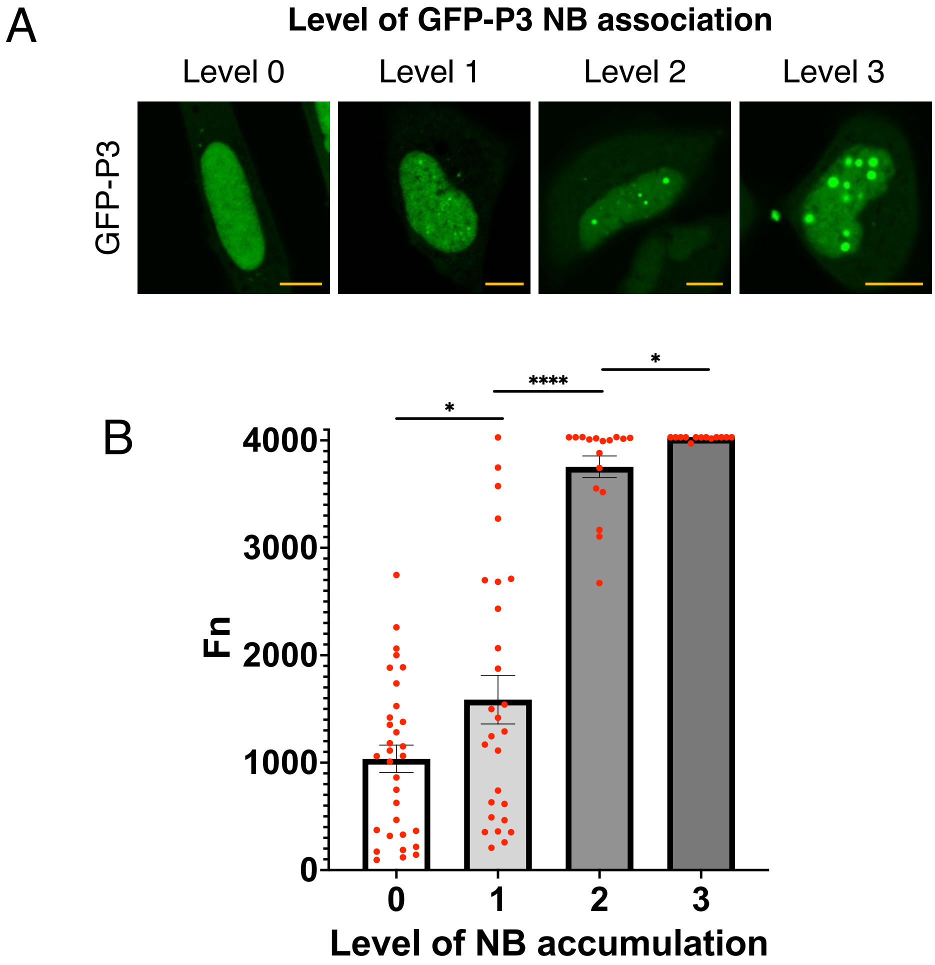

Supplementary Figure 3. The extent of P3 NB formation correlates with nuclear P3 fluorescence intensity.
(A) NB formation by GFP-P3 in HeLa cells varies between cells. Cells expressing GFP-P3 were categorized into four levels based on NB formation: no apparent NBs (Level 0), small NBs (Level 1), small and medium NBs (Level 2), and multiple large NBs (Level 3). Representative live-cell images of GFP-P3 at each level are shown. Scale bar: 10 µm (orange). (B) Image analysis was performed to assess (1) NB levels and (2) mean nuclear fluorescence intensity (*Fn*; nuclear fluorescence subtracting the background fluorescence) for each cell. Note that all images were acquired using the same microscope settings, so the fluorescence intensities of a proportion of bright nuclei were near or at saturation limit (4095, theoretical maximum); thus, the intensity may be higher than that measured. Data show mean *Fn* ± SEM (n = 89 cells from one independent assay). Statistical analysis was performed using unpaired two-tailed t-tests with Welch's correction. p-values: Level 0 vs Level 1 ($p = 0.0402$; *); Level 1 vs Level 2 ($p < 0.0001$; ****); Level 2 vs Level 3 ($p = 0.0162$; *). Source data are provided as a Source Data file.

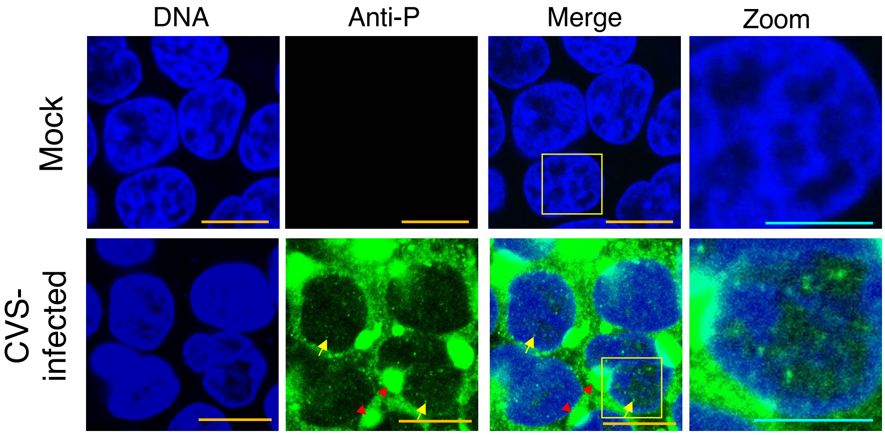

Supplementary Figure 4. Structures consistent with P protein nuclear bodies are formed during RABV infection.

(A) HEK-293 cells were either mock-infected or infected with the RABV strain CVS-11 (MOI = 2) and fixed at 48 h post-infection before immunofluorescence staining for the P protein (green). DNA was counterstained with Hoechst (blue). Yellow arrows indicate P protein-labelled structures consistent with NBs; red triangles highlight selected Negri bodies. Images are representative of >21 fields of view across two independent experiments. Quantification from one experiment showed that >95% of infected cells (128/134 cells) detectable P protein labelled NB structures. Scale bars: 10 µm (orange) for full-field images; 5 µm (light blue) for zoomed regions.

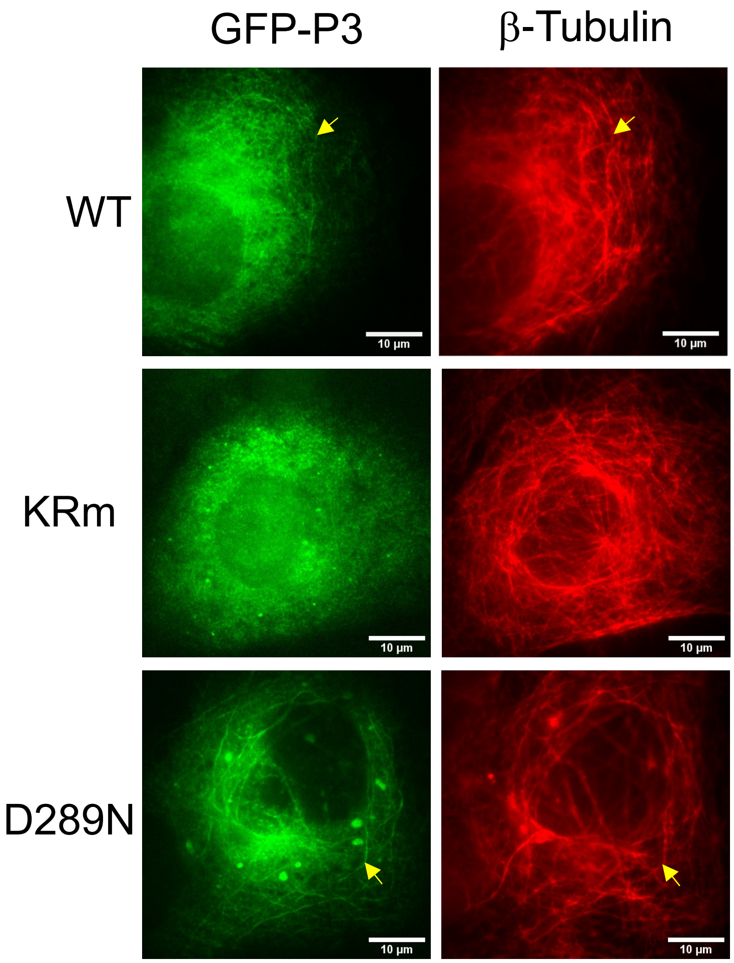

Supplementary Figure 5. Fluorescence images of cells used for dSTORM analysis. COS-7 cells transfected to express the indicated GFP-P3 protein were fixed and immunostained for β-tubulin (red). Cells expressing GFP-P3 were identified by epifluorescence microscopy (representative images shown) for *d*STORM analysis (see Figures 3E and 3F). Yellow arrows indicate example P3-associated MTs. Representative images from >4 fields of view across two independent experiments.

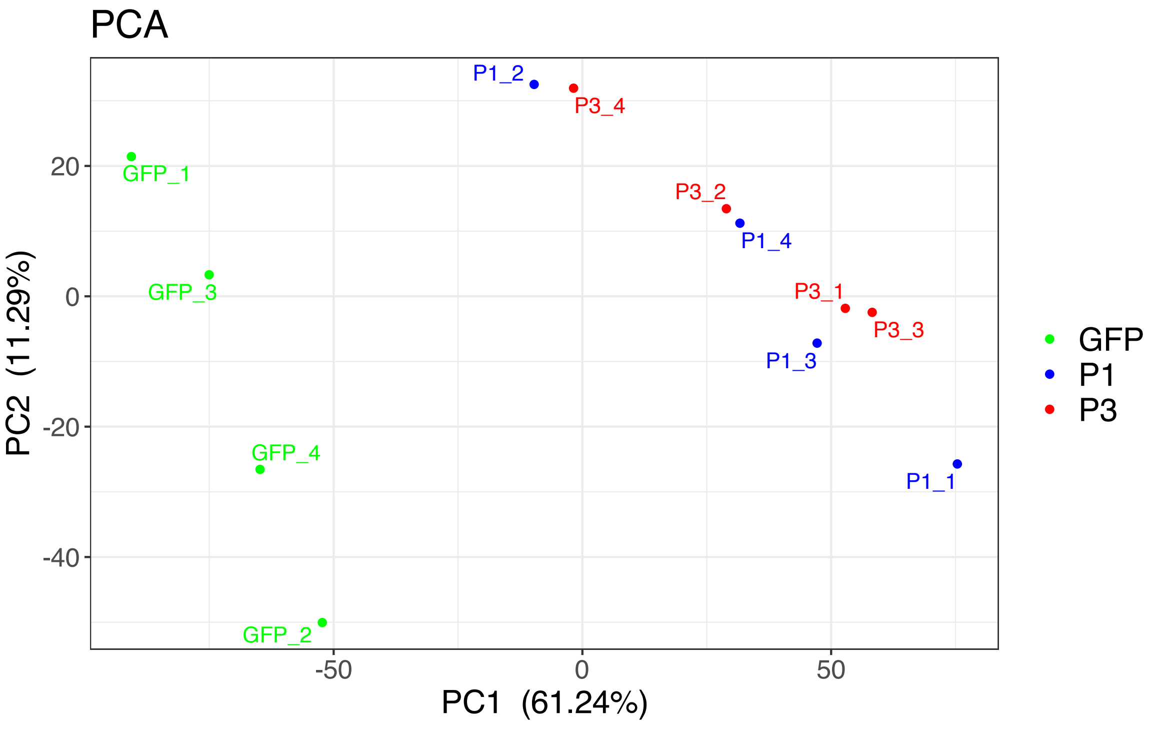

Supplementary Figure 6. PCA plots for GFP, GFP-P1 and GFP-P3 samples analyzed by IP-MS.

PCA plot showing that P1 and P3 interactomes identified by IP-MS cluster separately from GFP control interactomes (PCA shows four biological replicates, labelled 1-4, for each protein).

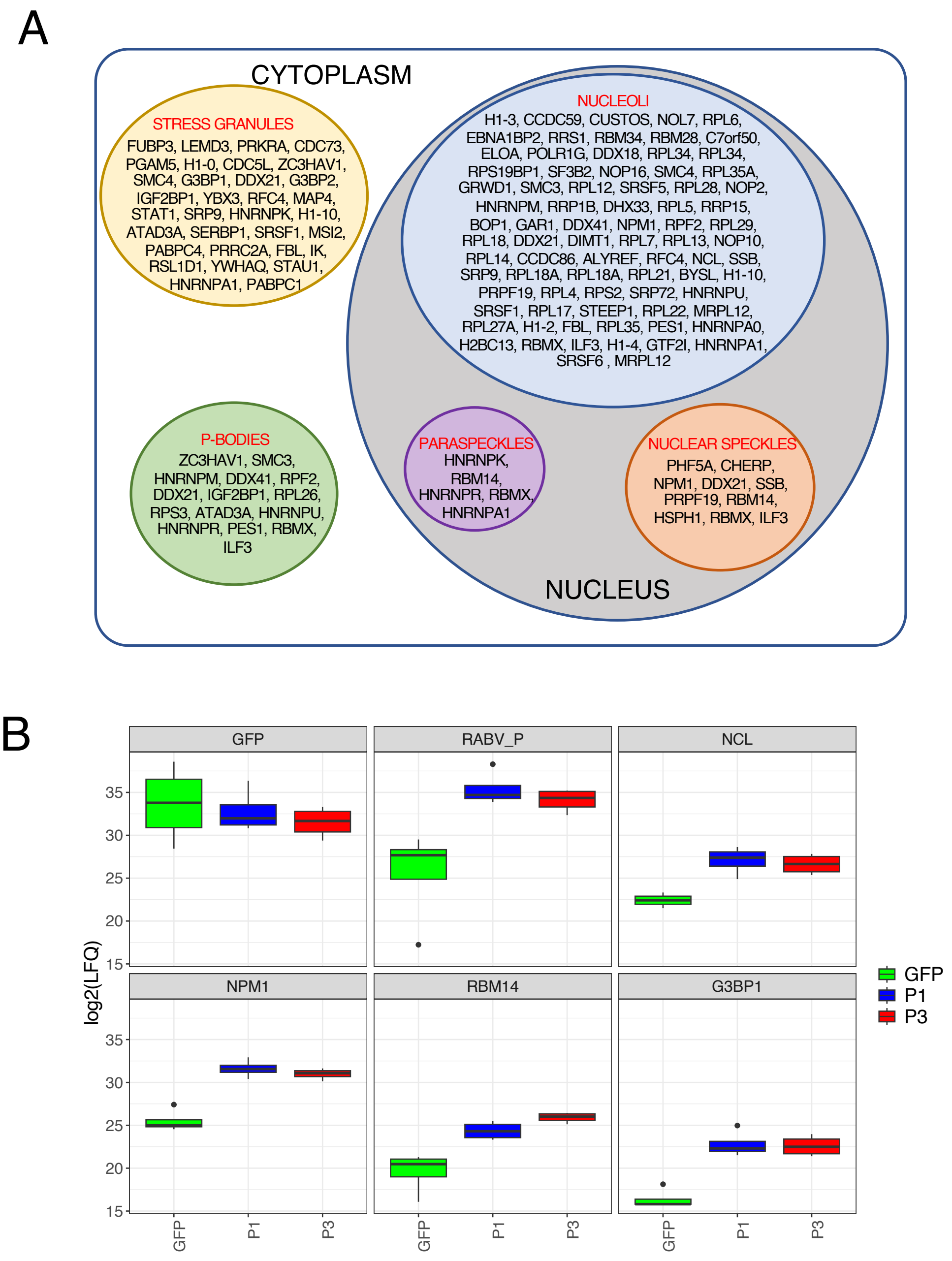

Supplementary Figure 7. RABV P3 associates with multiple proteins associated with MLOs.

(A) Schematic showing P3 protein interactors identified by IP-MS that are reported to be associated with cellular LLPS structures/MLOs. MLOs were determined by comparing proteins identified with localization reported in published datasets/lists (nucleoli, stress granules, paraspeckles, nuclear speckles and P-bodies). (B) Box plots showing quantitation (log2 LFQ intensity, n = 4 biological replicates) of proteins identified by IP-MS for each of GFP, GFP-P1 and GFP-P3.

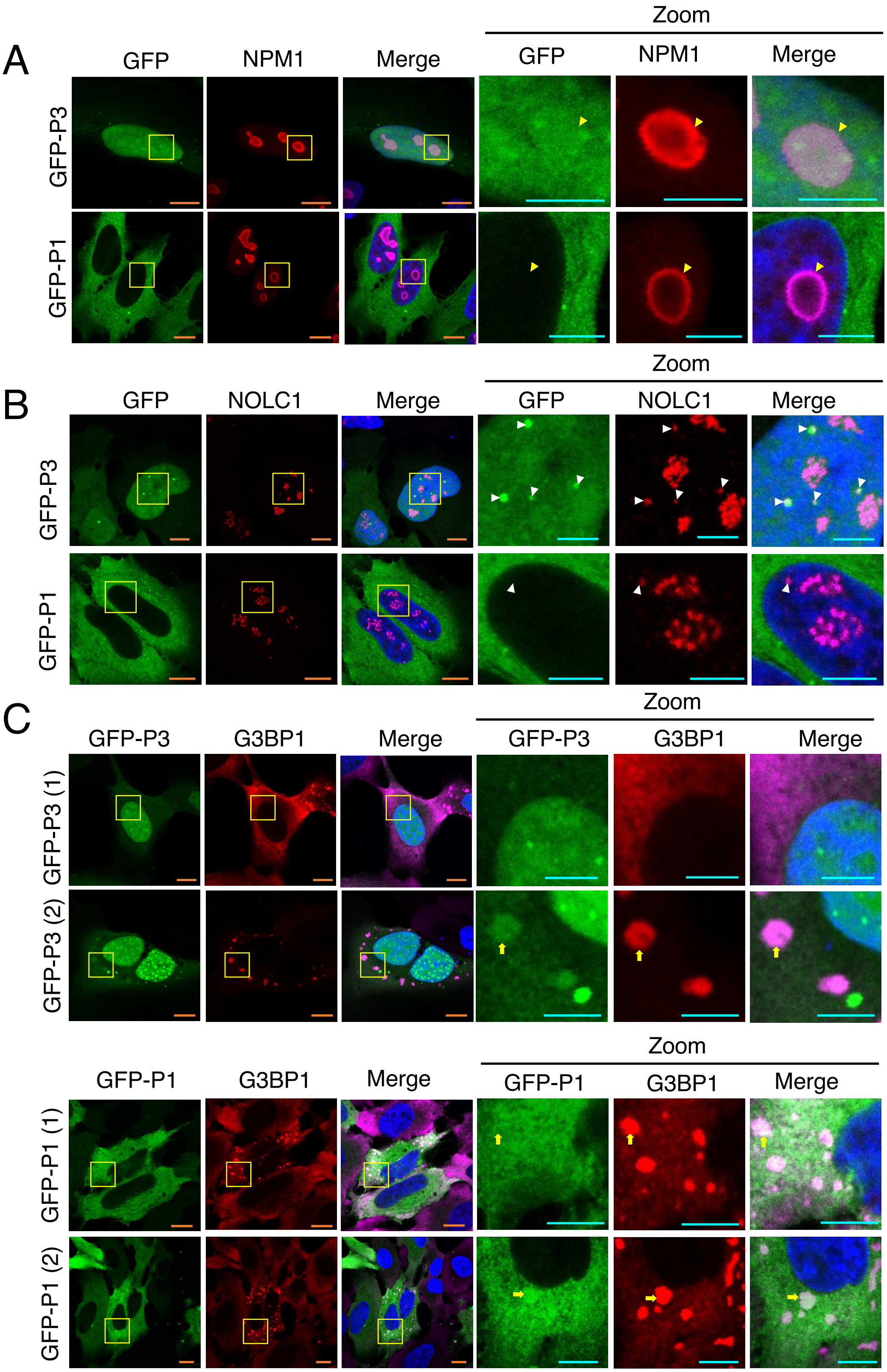

Supplementary Figure 8. Immunofluorescence analysis of selected interactors of P1 and P3 identified by IP-MS. HeLa cells transfected to express either GFP-P3 or GFP-P1 were fixed at 24 h p.t. and immunostained for (A) NPM1, (B) NOLC1, or (C) G3BP1, followed by imaging using CLSM. Two images are shown for G3BP1 (panels 1 and 2) to highlight differences in phenotypes observed. Yellow boxes indicate the regions magnified in the Zoom panels. Yellow arrowheads indicate nucleoli in NPM1 panels; white arrowheads indicate NOLC1-positive nuclear bodies; yellow arrows indicate cytoplasmic inclusions or stress granules formed by G3BP1. DNA/nuclei were counterstained with Hoechst (blue) in the merged images. Scale bars: 10 µm (orange) for full images and 5 µm (light blue) for zoom panels. UBF1, NPM1, NOLC1, and G3BP1 single-channel images are displayed in red, while merged images show these markers in magenta. Images are representative of ≥ 10 fields of view from one independent experiment.

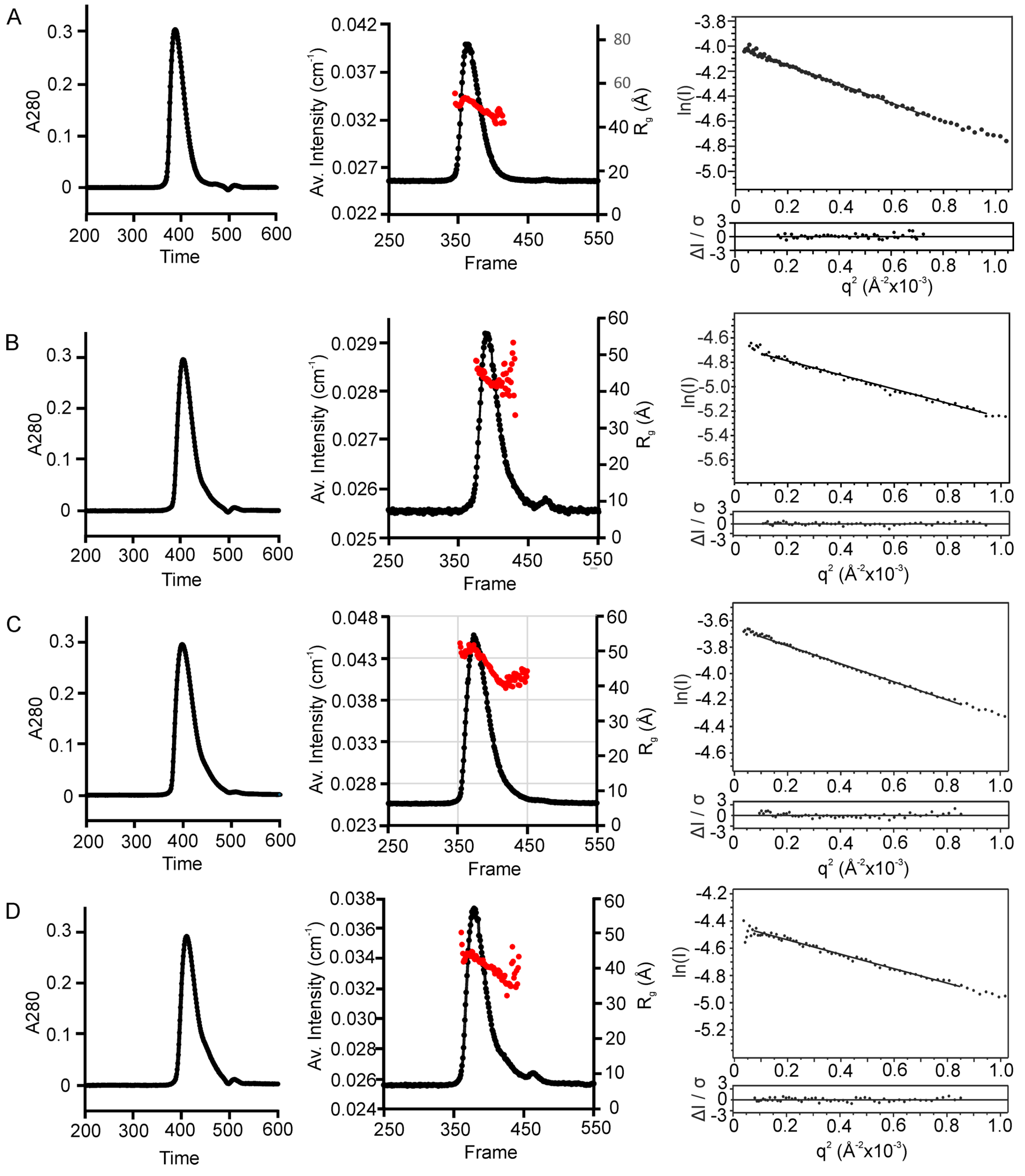

Supplementary Figure 9. Quality assessment of the SAXS data for P proteins. (A) P1 (B) wt-P3 (C) P3-KRm (D) P3-D289N. Left panels show SEC plots of UV absorbance at 280 nm versus time for the purification of the P proteins. Center panels show SEC-SAXS plots of average intensity versus frame (red circles show the respective Rg measured against frame). Right panels show the respective Guinier plots for the SAXS data in Figure 5.

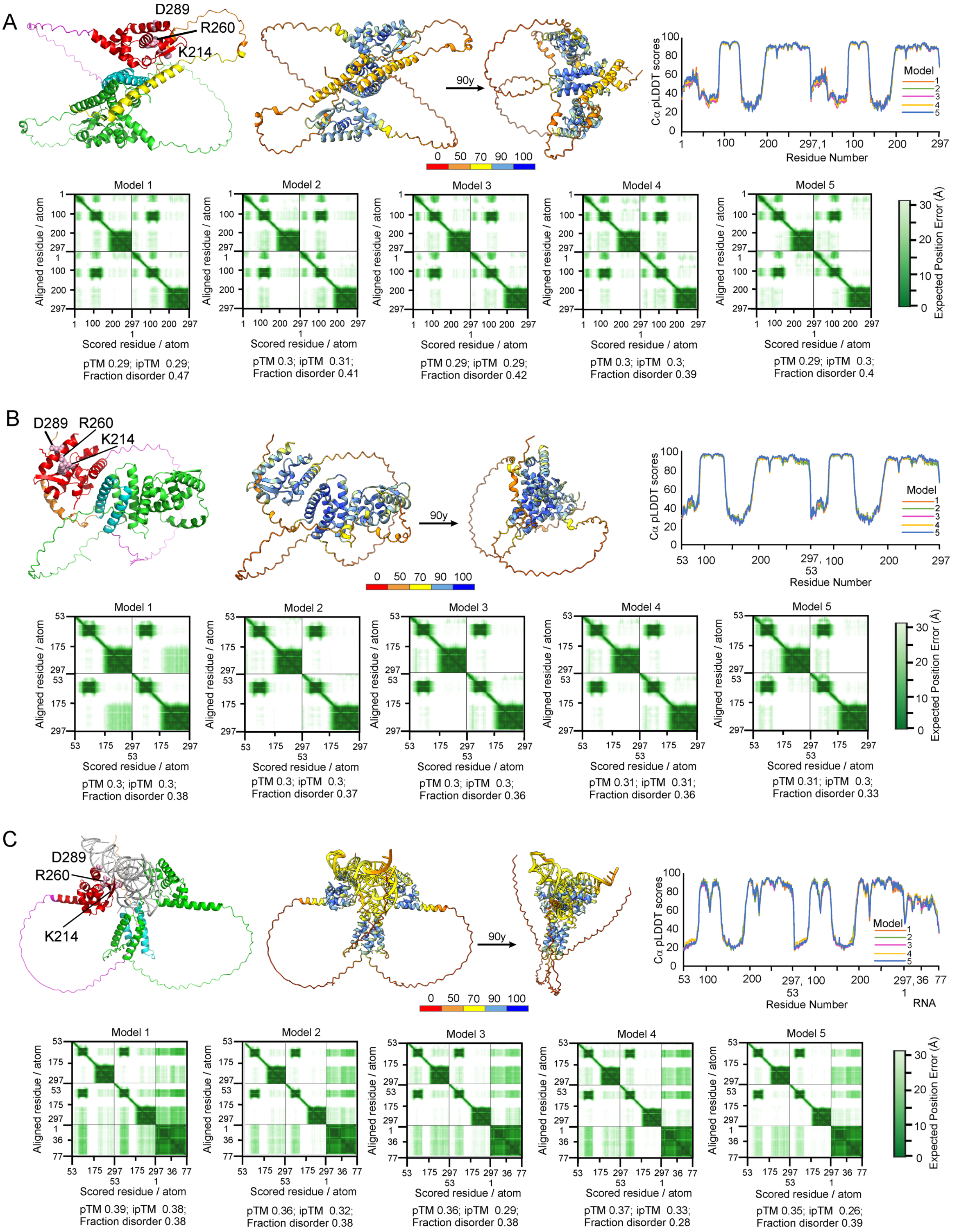

A
D289
R260
K214
90y
0 50 70 90 100
Cα pLDDT scores
Model
1
2
3
4
5
Residue Number
Model 1
Model 2
Model 3
Model 4
Model 5
Aligned residue / atom
Scored residue / atom
Expected Position Error (Å)
pTM 0.29; ipTM 0.29; Fraction disorder 0.47
pTM 0.3; ipTM 0.31; Fraction disorder 0.41
pTM 0.3; ipTM 0.29; Fraction disorder 0.42
pTM 0.3; ipTM 0.3; Fraction disorder 0.39
pTM 0.29; ipTM 0.3; Fraction disorder 0.4
B
D289
R260
K214
90y
0 50 70 90 100
Cα pLDDT scores
Model
Residue Number
Model 1
Model 2
Model 3
Model 4
Model 5
Aligned residue / atom
Scored residue / atom
Expected Position Error (Å)
pTM 0.3; ipTM 0.3; Fraction disorder 0.38
pTM 0.3; ipTM 0.3; Fraction disorder 0.37
pTM 0.3; ipTM 0.3; Fraction disorder 0.36
pTM 0.31; ipTM 0.31; Fraction disorder 0.36
pTM 0.31; ipTM 0.3; Fraction disorder 0.33
C
D289
R260
K214
90y
0 50 70 90 100
Cα pLDDT scores
Model
Residue Number
RNA
Model 1
Model 2
Model 3
Model 4
Model 5
Aligned residue / atom
Scored residue / atom
Expected Position Error (Å)
pTM 0.39; ipTM 0.38; Fraction disorder 0.38
pTM 0.36; ipTM 0.32; Fraction disorder 0.38
pTM 0.36; ipTM 0.29; Fraction disorder 0.38
pTM 0.37; ipTM 0.33; Fraction disorder 0.28
pTM 0.35; ipTM 0.26; Fraction disorder 0.39

Supplementary Figure 10. **Model prediction of conformational differences of P1 and P3, and of RNA binding to P3**.
Alphafold3 predicted models of (A) P1, (B) P3 and (C) P3 bound to torula RNA (tRNA). In each panel the structure of the first model is shown with one chain coloured green and the second chain coloured to indicate specific domains/regions (IDR1 residues 1-53 yellow, 53 to 88 orange; DD, cyan; IDR2, magenta; CTD, red). The residues mutated in this study (K214, R260, D289) are shown as pink spheres. Two orientations of the models are shown and colour-coded according to pLDDT scores based on the Cα (protein) or phosphorus atom (tRNA). Further, for all five models the pLDDT plots and 2D PAE maps of the relative positions of the domains and regions are shown. Under each PAE the predicted template model (pTM) score, predicted interface template model (ipTM) score, and predicted fractions of disorder are shown. Due to the high content of disorder the models have low confidence. Nevertheless, the models propose a mechanism whereby the NTD of P1 folds to interact with the CTD restricting the conformational flexibility of the CTD and the accessibility of K214 and R260 to potential ligands such as RNA. The truncation of the first 53 residues of IDR1 removes this interaction in P3, increasing the conformational flexibility of the CTD, exposing K214 and R260 for binding to RNA. This model proposes that the CTD of both protomers of P3 are required for binding RNA. While this model supports loss of binding for the KRm mutant of P3 it is not clear why D289N increases binding, suggesting other interactions, such as with the DD, are important. Source data are provided as a Source Data file.

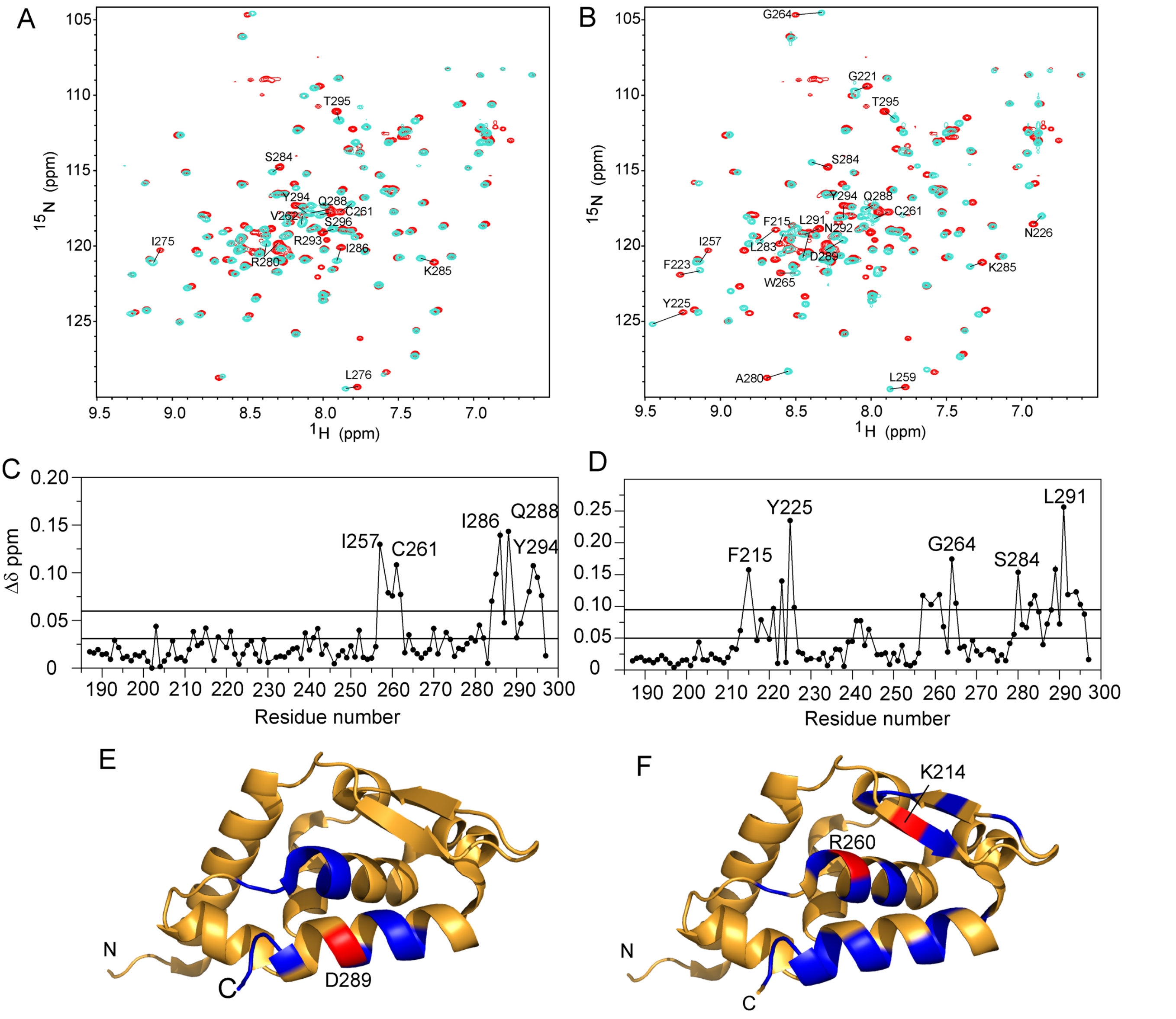

Supplementary Figure 11. Mutations D289N and K214/R260 (KRm) have minor effects on the structure of the P protein CTD. Overlay of the $^{1}H,^{15}N$ HSQC spectra of (A) wt (red) and D289N (cyan) CTD and (B) wt (red) and KRm (cyan) CTD. Assigned resonances that show the most significant shifts are labelled. The full spectrum of wt CTD is in Supplementary Figure 15. Plots of the average chemical shift differences ($\Delta\delta$ ppm) for ^{15}N and ^{1}HN were fitted to the equation $[(\Delta^{1}HN)^{2} + (0.15\times\Delta^{15}N)^{2})]^{1/2}$ for (C) D289N and (D) KRm versus wt CTD. Lines of mean and one standard deviation are shown. Nuclei that show greater than one SD difference are highlighted in blue on the structure of the CVS CTD (pdb: 1VYI) for (E) D289N and (F) KRm. Sites of mutations are coloured red. Source data are provided as a Source Data file.

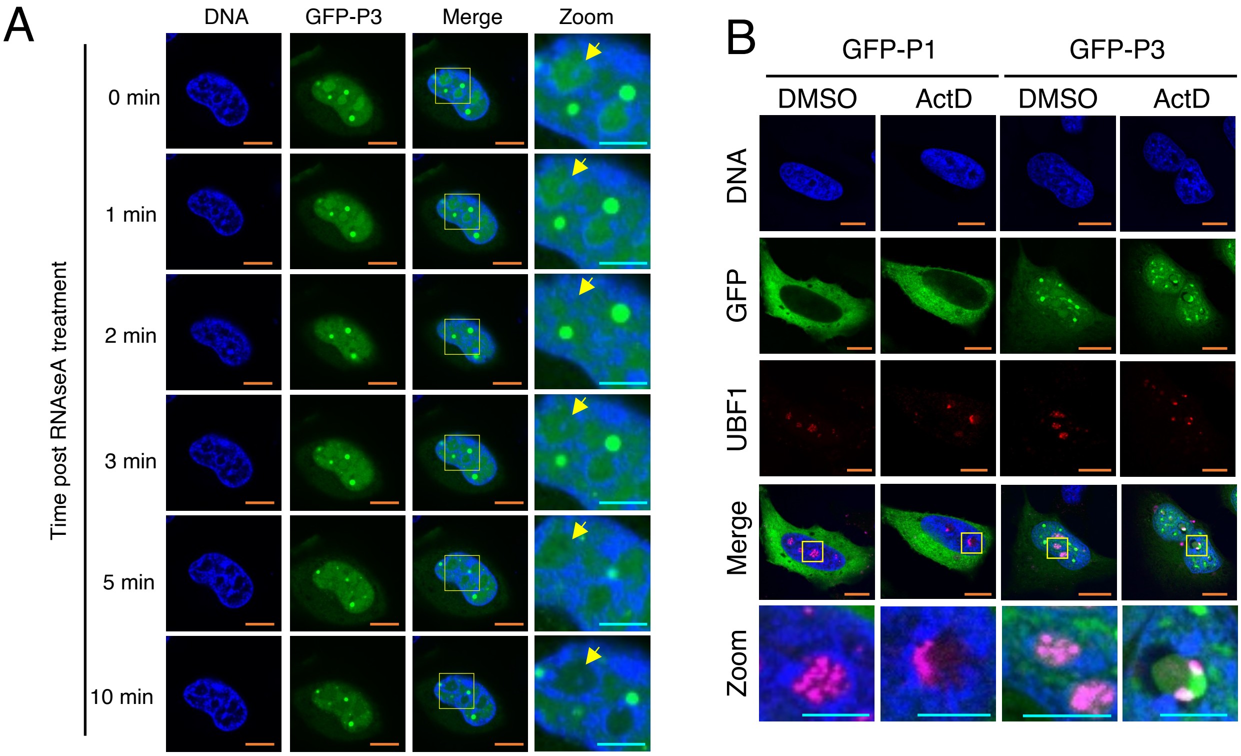

Supplementary Figure 12. Nucleolar targeting of P3 is affected by RNAse A and Actinomycin D treatment.

(A) HeLa cells transfected to express GFP–P3 were imaged live before (0 min) and after treatment (1–10 min) with RNase A. DNA/nuclei were visualized with Hoechst (blue). Merged images show DNA and GFP–P3. Yellow boxes indicate regions magnified in "Zoom" panels. Yellow arrows mark nucleoli. (B) HeLa cells expressing GFP–P1 or GFP–P3 were treated with DMSO (vehicle control) or Actinomycin D (ActD) for 4 h, then fixed at 24 h p.t. and immunostained for UBF1 (shown in red in single-channel images; magenta in merged panels). UBF1 served as a nucleolar marker, normally localizing to fibrillar centers (FCs) and the dense fibrillar component (DFC), but redistributing into nucleolar caps when rDNA transcription is inhibited (e.g., after ActD). Merged images show DNA, GFP–P1/P3, and UBF1. Yellow boxes indicate regions magnified in "Zoom" panels shown below. Scale bars: 10 µm (orange) for full-field images; 5 µm (light blue) for zoomed regions (applies to A and B).

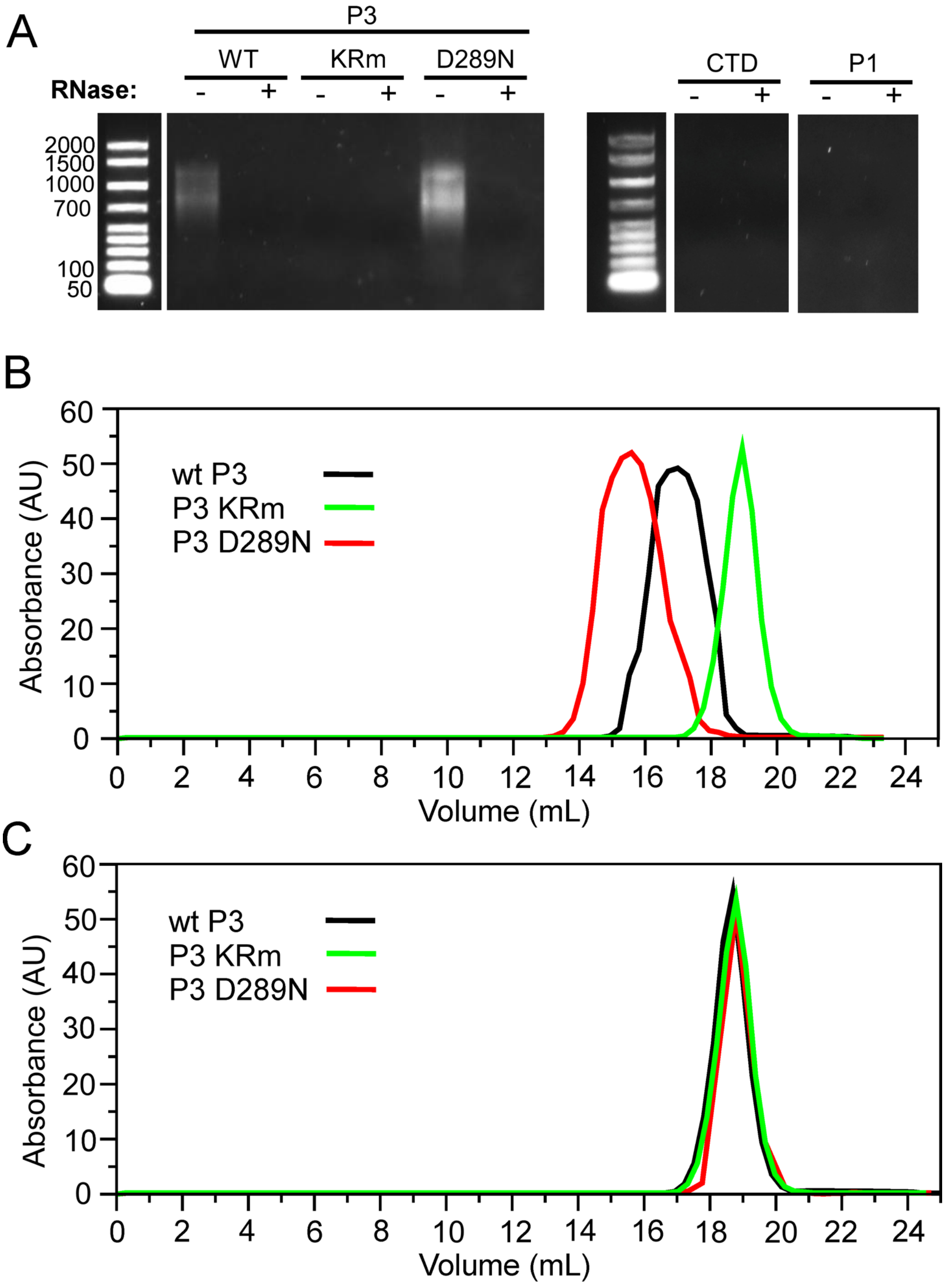

Supplementary Figure 13. Purified P3 but not P1 is associated with RNA. (A) Wt P3, P3-KRm, P3-D289N and P1 were expressed in *Escherichia coli* and purified in the presence or absence of RNAse A. In the absence of RNAse A, wt P3 and P3-D289N show association with nucleic acid, which is enhanced for P3-D289N, and lost in RNase A-treated samples. P3-KRm and P1 show no detectable interaction with RNA. Intervening lanes of the agarose gels have been removed for viewing relevant protein/RNA interactions. Full and uncropped gels are shown in source data file. (B, C) Size-exclusion chromatography of wt P3 (black) and P3-D289N (red) shows that these proteins elute at a lower volume than P3-KRm (green) in the absence of RNAse A (B), and wt P3 and P3-D289N samples treated with RNase A (C). P3-KRm (green) shows a similar chromatogram in the absence (B) and presence of RNase A (C).

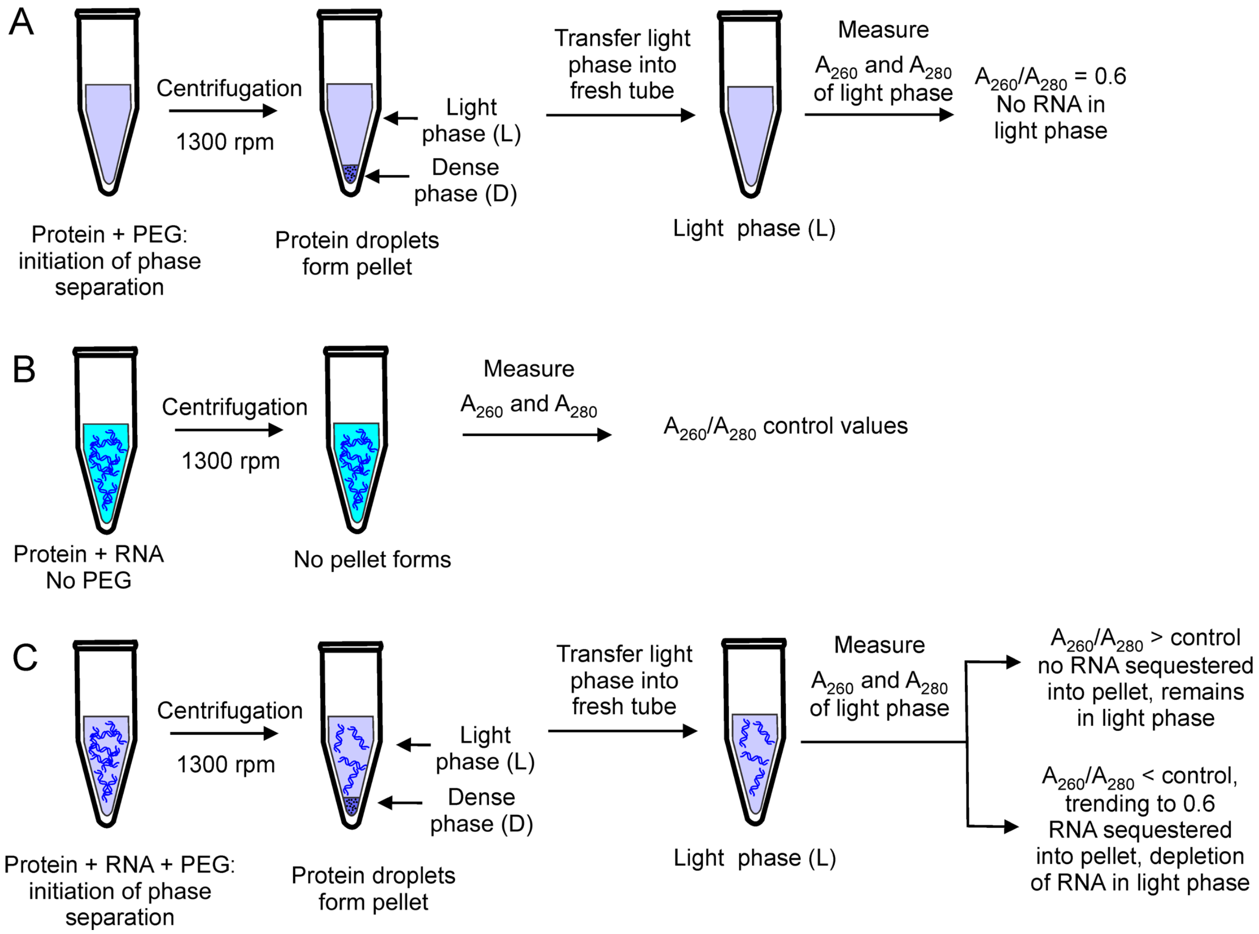

Supplementary Figure 14. Schematic showing procedure for assay to measure the sequestration of RNA into liquid bodies. To assess the ability of P protein to bind and sequester RNA we developed an assay using measurement of A_{260}/A_{280} of the light phase in the absence and presence of 10% PEG and RNA. Samples were prepared at 100 µM protein. Under phase separation conditions (10% PEG) 60% of protein is estimated to be in the dense phase. (A) Under phase separation conditions the light phase for all P protein tested in the absence of RNA shows an A_{260}/A_{280} ~0.6. (B) To obtain control values, protein is mixed at different ratios with RNA (without PEG) and centrifuged before measurement of A_{260}/A_{280} which, at all concentrations of RNA, is > 0.6. (C) The assay is conducted as in (B), but with 10% PEG to induce phase separation. Following centrifugation, the A_{260}/A_{280} of the light phase is measured. If RNA is sequestered into the dense phase, A_{260}/A_{280} of the light phase will be reduced compared with the control (B) and will approach 0.6. If RNA is not sequestered and so remains in the light phase, the A_{260}/A_{280} will be increased compared with the control (B) and will not approach 0.6.

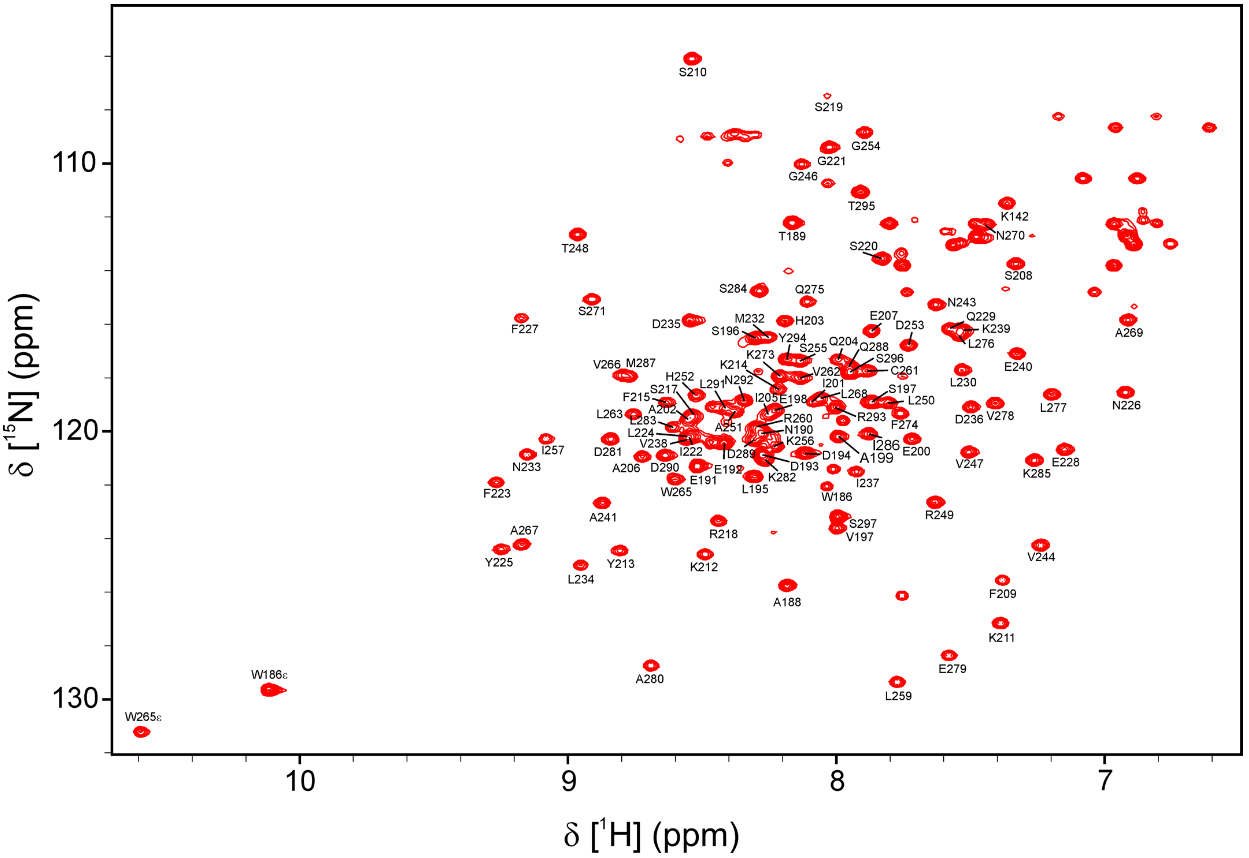

Supplementary Figure 15. $^{1}H,^{15}N$ HSQC of wild-type CTD of CVS P protein. The ^{1}HN, ^{15}N, $^{13}C\alpha$ and $^{13}C\beta$ resonances of 500 μM CTD were assigned by triple resonance NMR spectroscopy at pH 6.8 and 20 °C. 98% of the non-proline ^{1}HN and ^{15}N resonances were assigned with only Lys231 and Lys272 not identified.

Supplementary Table 1: SAXS results for CVS wt P1, P3, P3-KRm and P3-D289N.

a) Sample details:	P1	P3	P3-KRm	P3-D289N
Organism	Rabies virus (strain CVS-11) (RABV)			
Source	Recombinant expression from *Escherichia coli* (BL21, DE3)			
Description: sequence	Uniprot ID P22363, N-terminus has additional Glycine			
Extinction coefficient at 280 nm [A280, 0.1%(w/v)]	0.756	0.913	0.918	0.913
$\bar{v}$ from chemical composition (cm^3g^{-1})	0.734	0.734	0.734	0.734
Particle contrast from sequence and solvent constituents, $\Delta\rho$ ($\rho_{protein}$ - $\rho_{solvent}$; 10^{10} cm^{-2})	2.83	2.80	2.79	2.79
Molecular Mass (kDa)	67.3	56.1	55.8	56.1

SEC-SAXS parameters:	
Column	Superose 6 5×150
Flow rate (mL/min)	0.4
Concentration (mg/mL)	5
Injection volume (µL)	50
Average conc. (mg/mL)	3.02
Solvent	25 mM HEPES, pH 7.4, 150 mM NaCl, 1 mM TCEP, 0.1% sodium azide

b) SAXS data collection parameters:	
Instrument/source	Australian Synchrotron SAXS/WAXS beamline equipped with Pilatus 1M detector and sheath-flow cell for SEC-SAXS.
Wavelength (Å)	1.0332
Beam energy (kEv)	12
Beam size (µm)	250 × 130
Sample-to-detector distance (mm)	3256
q ($Å^{-1}$)	0.005 – 0.334
Absolute scaling method	Comparison with scattering from 1 mm pure water
Normalization	To transmitted intensity from beamstop counter.
Exposure time	Continuous 1 s data frame measurements of SEC-SAXS elution
Sample temperature (K)	295
Sample configuration	SEC–SAXS with sheath flow cell, effective sample path length 0.49mm

c) Software employed	
SAXS data reduction	$I(q)$ vs q using Scatterbrain 2.8.2, SEC-SAXS solvent subtraction using *CHROMIXS* from *ATSAS* 3.2.1
Basic analysis (Guinier, *P(r)*, molecular mass)	*PRIMUSqt* from *ATSAS* 3.2.1
Ab initio modelling	*DAMMIN* from *ATSAS* 3.2.1
Calculation of theoretical intensities	*CRYSOL* from *ATSAS* 3.2.1
Atomic structure (hybrid) modelling	*CORAL* from *ATSAS* 3.2.1
Calculation of ε from sequence	https://web.expasy.org/protparam[1]
Calculation of $\Delta\bar{\rho}$ and $\bar{\upsilon}$ values from chemical composition	SASSIE Contrast Calculator (SCC)[2]

d) Structural parameters				
Mass from V_c (kDa) (expected mass, ratio to expected in brackets)	88.1 (67.3, 1.3)	62.5 (56.1, 1.1)	65.8 (55.8, 1.18)	61.3 (56.1, 1.09)
Guinier analysis				
R_g (Å)	47.35± 0.24	41.79 ± 0.31	45.88 ± 0.32	39.42 ± 0.22
I(0) (cm^{-1})	0.018 ± 0.00006	0.01 ± 0.00004	0.026 ± 0.00007	0.012 ± 0.00004
qR_g min,max	0.45, 1.29	0.44, 1.28	0.45, 1.22	0.33, 1.29
P(r) analysis				
R_g (Å)	49.25 ± 0.02	41.60 ± 0.01	46.12 ± 0.03	40.01 ± 0.01
D_{max} (Å)	170.11	144.38	158.86	133.22
Porod volume (Å^3)	217946.00	13777.40	170341.00	122475.01

e) Ensemble Optimization Methodology				
Starting structure	Homology model of dimerization domain (DD) of CVS P protein obtained using PDB ID: 3L32 (China/MRV strain) and C-terminal domain (CTD) of CVS P protein, PDB ID: 1VYI			
Symmetry assumptions	P2 for DD and was kept fixed as in the original PDB)			
No. of models in the pool	*RANCH* was used to generate a pool of 10000 models			
Theoretical intensity	*Crysol* was used to calculate the theoretical intensity along with R_g and D_{max}			
Genetic Algorithm	Yes, once, using *GAJOE*			
χ2 range	0.96	0.92	1.34	1.04

f) Deposition codes				
SASBDB Code	SASDRB6	SASDRX4	SASDRZ4	SASDRY4

1. Gasteiger E., Hoogland C., Gattiker A., Duvaud S., Wilkins M.R., Appel R.D., Bairoch A. (2005) Protein Identification and Analysis Tools on the Expasy Server. (In) John M. Walker (ed): The Proteomics Protocols Handbook. pp. 571-607. Humana Press.

2. Sarachan, K., Curtis, J.E., Krueger, S. (2013) Small-angle scattering contrast calculator for protein and nucleic acid complexes in solution. J. Appl. Cryst. 46, 1889-1893.

Supplementary Table 2. Hydrogen-deuterium exchange for wt P protein CTD and mutants K214/R260 (KRm) and D289N.[a]

Residue	Secondary structure	Wild Type (k_{ex} x 10^{-3} min^{-1})	D289N (k_{ex} x 10^{-3} min^{-1})	δΔG ($kJ.mol^{-1}$)	KRm (k_{ex} x 10^{-3} min^{-1})	δΔG ($kJ.mol^{-1}$)
A202	α1	1.6 ± 0.3	1.3 ± 0.6	0.5	3.7 ± 0.4	-2.1
F215	β1	1.6 ± 0.3	2.3 ± 0.6	-0.9	7.4 ± 0.7	-3.8
F227	3^{10} helix	4.7 ± 0.3	4.1 ± 1.5	0.3	19.5 ± 2.5	-3.5
E240	α2	1.2 ± 0.3	1.3 ± 0.4	-0.2	2.6 ± 0.6	-1.9
V262	α4	0.8 ± 0.1	1.0 ± 0.3	-0.6	2.2 ± 1.3	-2.5
L277	α5	2.2 ± 0.3	2.0 ± 0.3	0.2	4.4 ± 2.0	-1.7

[a] Exchange rates of peptide HN groups with ^2H_2O at pH 6.8 and 25 °C. Rates and free energy differences of exchange (δΔG) are given example protons throughout the protein.
