# Supplementary material for: Conformational dynamics, RNA binding, and phase separation regulate the multifunctionality of rabies virus P protein
Source: Nat Commun. 2025 Oct 29;16:9491. doi: 10.1038/s41467-025-65223-y (PMC12589655; doi:10.1038/s41467-025-65223-y)
Supplement: Supplementary file 2 — Description of Additional Supplementary Files [file 41467_2025_65223_MOESM2_ESM.pdf]

Description of Additional Supplementary Files

Supplementary Data 1

Proteins identified by immunoprecipitation–mass spectrometry (IP-MS) of GFP-P1 and GFP-P3 in HEK-293T cells

This file contains quantitative proteomics data from GFP-Trap immunoprecipitation coupled with LC-MS/MS (IP-MS) analysis of GFP-P1, GFP-P3, and GFP control samples expressed in HEK-293T cells. The dataset lists all proteins identified, their quantitative values, statistical significance, enrichment relative to GFP control. Statistical significance was assessed using two-sided, unpaired t-tests in Perseus on $\log_2$-transformed LFQ intensities, with missing values imputed. Hits were considered significant at $p < 0.05$ and $\log_2$ fold change > 1.

The complete proteomics dataset has been deposited to the ProteomeXchange Consortium via the PRIDE partner repository with the dataset identifier PXD064752.

Supplementary Data 2

Quantitative crosslinking mass spectrometry results of protein samples P1, P3, P3_D289N and P3_KRm.

Unique cross-linked peptide abundance for each protein sample was measured in 4 biological replicates (reported under column names 'Abundance: <protein name> (1-4)'. Median abundance and coefficient of variation (CV%) were calculated for the peptides of each protein. Median abundance ratios for P1/P3, P3_D289N/P3 and P3_KRm/P3 were calculated using the pairwise ratios approach for each crosslinked peptide pair, where the geometric median of all combinations of ratios from all the replicates is calculated. When a peptide abundance was detected in less than three biological replicates per group, the peptide was eliminated from further analysis using xiVIEW. p-values were calculated using a two-tailed t test (Wilcoxon test) by comparing the median abundance ratios of each peptide per group to 1. All cross-linked peptides comprise unique lys-lys cross-links.

Supplementary Movie 1

Dynamics of P3-associated nuclear bodies in live cells

Time-lapse confocal microscopy of live HeLa cells expressing GFP-P3, showing formation, movement, and fusion of P3-positive nuclear bodies over time. The movie illustrates the liquid-like, dynamic behavior of P3 nuclear bodies characteristic of LLPS-driven membraneless organelles, as described in Figure 2A.
