# Supplementary material for: Conformational dynamics, RNA binding, and phase separation regulate the multifunctionality of rabies virus P protein
Source: Nat Commun. 2025 Oct 29;16:9491. doi: 10.1038/s41467-025-65223-y (PMC12589655; doi:10.1038/s41467-025-65223-y)
Supplement: Supplementary file 6 — Reporting Summary [file 41467_2025_65223_MOESM6_ESM.pdf]

nature portfolio

Corresponding author(s): A/Prof. Greg Moseley
Prof. Paul Gooley
Dr. Stephen Rawlinson

Last updated by author(s): Sep 26, 2025

Reporting Summary

Nature Portfolio wishes to improve the reproducibility of the work that we publish. This form provides structure for consistency and transparency in reporting. For further information on Nature Portfolio policies, see our Editorial Policies and the Editorial Policy Checklist.

Statistics

For all statistical analyses, confirm that the following items are present in the figure legend, table legend, main text, or Methods section.

n/a	Confirmed	
☐	☒	The exact sample size (n) for each experimental group/condition, given as a discrete number and unit of measurement
☐	☒	A statement on whether measurements were taken from distinct samples or whether the same sample was measured repeatedly
☐	☒	The statistical test(s) used AND whether they are one- or two-sided<br>*Only common tests should be described solely by name; describe more complex techniques in the Methods section.*
☐	☒	A description of all covariates tested
☒	☐	A description of any assumptions or corrections, such as tests of normality and adjustment for multiple comparisons
☐	☒	A full description of the statistical parameters including central tendency (e.g. means) or other basic estimates (e.g. regression coefficient) AND variation (e.g. standard deviation) or associated estimates of uncertainty (e.g. confidence intervals)
☐	☒	For null hypothesis testing, the test statistic (e.g. F, t, r) with confidence intervals, effect sizes, degrees of freedom and P value noted<br>*Give P values as exact values whenever suitable.*
☒	☐	For Bayesian analysis, information on the choice of priors and Markov chain Monte Carlo settings
☒	☐	For hierarchical and complex designs, identification of the appropriate level for tests and full reporting of outcomes
☒	☐	Estimates of effect sizes (e.g. Cohen's d, Pearson's r), indicating how they were calculated

Our web collection on statistics for biologists contains articles on many of the points above.

Software and code

Policy information about availability of computer code

Data collection	Confocal microscopy images were acquired using a Leica SP5 or Nikon C1 microscope with NIS-Elements AR software (v4.13.01, Build 916) or a Zeiss LSM 800 microscope running ZEN Blue (version as per facility). Live-cell imaging for nuclear body (NB) fusion assays was performed on a Nikon Ti2 microscope equipped with an environmental chamber. Super-resolution dSTORM imaging was conducted using Micro-Manager (open source), and dSTORM image reconstruction was performed with RapidSTORM (open source). Confocal and gel image analyses were performed using Fiji/ImageJ (v2.1.0/1.53c, open source).<br>For NMR spectroscopy, data acquisition was conducted using TopSpin (v3.2.7, Bruker). SAXS data were collected at the Australian Synchrotron using Scatterbrain (v2.8.2) and analyzed with the ATSAS package (v3.2.1).<br>Structural predictions were generated using AlphaFold (v3.0.1), and structures were visualized with UCSF ChimeraX (v1.7) and PyMOL (v2.5).<br>RNA-binding and phase separation data were analyzed using turbidity assays and phase diagrams processed with Microsoft Excel and GraphPad Prism (v10).<br>LC-MS/MS data were acquired on an Orbitrap Ascend mass spectrometer (Thermo Fisher Scientific, USA) controlled by Xcalibur (v4.x), processed using MaxQuant (v2.6.1.0) and Perseus (v2.0.11), and XL-MS quantification was performed using Proteome Discoverer (v2.3).

Data analysis	Statistical analyses were performed using GraphPad Prism (v10) unless otherwise specified. Confocal image quantification, including fluorescence intensity measurements (Fn/c, Fnu/n), was performed using Fiji/ImageJ (v2.1.0/1.53c, open source).<br>Small-angle X-ray scattering (SAXS) data were analyzed using the ATSAS package (v3.2.1), with parameters reported in Supplementary Table 1. Raw immunoprecipitation mass spectrometry (IP-MS) data were processed using MaxQuant (v2.6.1.0) against the human UniProt/Swiss-Prot database, and downstream statistical analyses were performed using Perseus (v2.0.11).<br>Cross-linking mass spectrometry (XL-MS) data were quantified using the Minora Feature Detector and Precursor Ion Quantifier nodes within Proteome Discoverer (v2.3, Thermo Fisher Scientific). Data were normalized to the corresponding GFP control, and differential abundance was assessed at the cross-linked residue-pair level.

For manuscripts utilizing custom algorithms or software that are central to the research but not yet described in published literature, software must be made available to editors and reviewers. We strongly encourage code deposition in a community repository (e.g. GitHub). See the Nature Portfolio guidelines for submitting code & software for further information.

Data

Policy information about availability of data

All manuscripts must include a data availability statement. This statement should provide the following information, where applicable:

- Accession codes, unique identifiers, or web links for publicly available datasets
- A description of any restrictions on data availability
- For clinical datasets or third party data, please ensure that the statement adheres to our policy

The XL-MS data and IP-MS have been deposited to the ProteomeXchange Consortium via the PRIDE103 partner repository under accession codes PXD064535 [http://proteomecentral.proteomexchange.org/cgi/GetDataset?ID=PXD064535] and PXD064752 [http://proteomecentral.proteomexchange.org/cgi/GetDataset?ID=PXD064752]. The NMR assignments of ^15N, ^1HN, ^13Cα, and ^13Cβ of CVS wt CTD have been deposited at the Biological Magnetic Resonance Data Bank (BMRB) under accession code 53374 [https://bmrb.io/data_library/summary/index.php?bmrbId=53374]. Small-angle X-ray scattering (SAXS) data have been deposited in the Small Angle Scattering Biological Data Bank (SASBDB) under accession codes SASDRB6 [https://www.sasbdb.org/data/SASDRB6/] (P1), SASDRX4 [https://www.sasbdb.org/data/SASDRX4/] (P3), SASDRZ4 [https://www.sasbdb.org/data/SASDRZ4/] (P3-KRm), and SASDRY4 [https://www.sasbdb.org/data/SASDRY4/] (P3-D289N). All other data supporting the findings of this study are available within the paper, its Supplementary Information, and the accompanying Source Data file.

Research involving human participants, their data, or biological material

Policy information about studies with human participants or human data. See also policy information about sex, gender (identity/presentation), and sexual orientation and race, ethnicity and racism.

Reporting on sex and gender	N/A
Reporting on race, ethnicity, or other socially relevant groupings	N/A
Population characteristics	N/A
Recruitment	N/A
Ethics oversight	N/A

Note that full information on the approval of the study protocol must also be provided in the manuscript.

Field-specific reporting

Please select the one below that is the best fit for your research. If you are not sure, read the appropriate sections before making your selection.

- [x] Life sciences
- [ ] Behavioural & social sciences
- [ ] Ecological, evolutionary & environmental sciences

For a reference copy of the document with all sections, see nature.com/documents/nr-reporting-summary-flat.pdf

Life sciences study design

All studies must disclose on these points even when the disclosure is negative.

Sample size	No formal statistical methods were used to predetermine sample size. For quantitative cell-based assays, sample sizes (typically ≥ 3 independent biological replicates with ≥ 10–40 cells per replicate) were chosen based on established practice in the field and previous publications to ensure reproducibility and sufficient power to detect biologically meaningful differences. For biochemical assays (e.g., immunoblots, IP-MS, XL-MS), replicate numbers were based on feasibility and are consistent with similar studies in the literature. The robustness of our findings is supported by the consistency of results across independent experiments, as reflected in the statistical significance and low variability reported in the figures.
Data exclusions	No data excluded
Replication	All key findings were successfully replicated in ≥2 independent experiments, with consistent results.

Randomization	Not applicable. No organisms or subjects that require randomization were analysed.
Blinding	Not applicable. No organisms or subjects that require blinding were analysed.

Reporting for specific materials, systems and methods

We require information from authors about some types of materials, experimental systems and methods used in many studies. Here, indicate whether each material, system or method listed is relevant to your study. If you are not sure if a list item applies to your research, read the appropriate section before selecting a response.

Materials & experimental systems

n/a	Involved in the study
☐	☒ Antibodies
☐	☒ Eukaryotic cell lines
☒	☐ Palaeontology and archaeology
☒	☐ Animals and other organisms
☒	☐ Clinical data
☒	☐ Dual use research of concern
☒	☐ Plants

Methods

n/a	Involved in the study
☒	☐ ChIP-seq
☒	☐ Flow cytometry
☒	☐ MRI-based neuroimaging

Antibodies

Antibodies used	Antibodies used in this study include NCL (CST, Cat# 14574; IB 1:1000), NPM1 (ThermoFisher, Cat# 32-5200; IB 1:2000), NOLC1 (ProteinTech, Cat# 11815-1-AP; IB 1:2000; Abcam, Cat# Ab204318, IF 1:50), RBM14 (Bethyl Laboratories, Cat# A300-331A; IB 1:2000, IF 1:50), SFPQ (Abcam, Cat# Ab11825; IB 1:1000), NONO (Abcam, Cat# Ab133574; IB 1:2000, IF 1:250), G3BP1 (Santa Cruz, Cat# sc-81940; IB 1:200, IF 1:100), Treacle (ProteinTech, Cat# 11003-1-AP; IB 1:2000, IF 1:100), Coilin (Santa Cruz, Cat# sc-55594; IF 1:200), SMN (BD Biosciences, Cat#610646, IF 1:1000), PML (Santa Cruz, Cat# sc-966; IF 1:50), GFP (Abcam, Cat# Ab6556; IB 1:5000),-tu [illegible] (Sigma, Cat# T8328; IB 1:5000; IF 1:1000), UBF1(Abcam, Cat# Ab244287; IF 1:500), Rabies P-protein (Cat#MBS1489481; IF 1:200 for untagged P-protein staining). Secondary antibodies (1:10,000 dilution) used for IB were goat anti-rabbit (Merck, Cat#AP307P) and goat anti-mouse (Merck, Cat# AP308P) IgG horse radish peroxidase (HRP)-conjugated-antibodies. Secondary antibodies (1:1000 dilution) used for IF were goat anti-rabbit Alexa Fluor 568 (Cat #A-11011) and goat anti-mouse Alexa Fluor 568 (Cat #A-11004), purchased from Thermo Fisher Scientific. To inhibit CRM1-mediated nuclear export, cells were treated with leptomycin-B (LMB, Cell Signaling, Cat#9676; 2.8 ng/mL) for 3 h prior to analysis. All antibodies used are commercially sourced and validated by the manufacturer for the stated application (IB, IF). Where available, validation information was confirmed from the supplier's datasheet. Lot numbers were not consistently tracked and are therefore not reported.
Validation	All antibodies used (except anti-P used for infection work (see below)) are commercial antibodies that have been validated by the company. In addition, all antibodies used for IP/Western analysis included whole-cell lysate controls, to validate expected size of proteins. anti-P antibody (M241882, diluted 1:10; a kind gift from Dr. Christine Fehlner-Gardiner, Centre of Expertise for Rabies, Canadian Food Inspection Agency) was used for infection work. It has been validated and is published (ref 82).

Eukaryotic cell lines

Policy information about cell lines and Sex and Gender in Research

Cell line source(s)	HeLa cells were obtained from ATCC (CCL-2)<br>HEK-293T cells were obtained from ATCC (CRL-3216)<br>COS-7 cells were obtained from ATCC (CRL-1651)
Authentication	Cell lines not authenticated
Mycoplasma contamination	Cells used in this study tested negative for mycoplasma.
Commonly misidentified lines<br>(See ICLAC register)	no commonly misidentified cell lines were used

Plants

Seed stocks	N/A
Novel plant genotypes	N/A
Authentication	N/A
